# Feeding Behavior of the European Brown Hare (*Lepus europaeus*, Leu2 Haplotype) on Pianosa Island: Insights into the Absence of Trophic Competition

**DOI:** 10.3390/vetsci12060546

**Published:** 2025-06-03

**Authors:** Pierangelo Freschi, Simonetta Fascetti, Francesco Riga, Marco Zaccaroni, Francesca Giannini, Emilia Langella, Carlo Cosentino

**Affiliations:** 1 Department of Agricultural, Forestry, Food and Environmental Sciences (DAFE), University of Basilicata, 85100 Potenza, Italy; simonetta.fascetti@unibas.it (S.F.); emilia.langella@unibas.it (E.L.); carlo.cosentino@unibas.it (C.C.); 2 Italian Institute for Environmental Protection and Research (ISPRA), 00144 Rome, Italy; francesco.riga@isprambiente.it; 3 Department of Biology (BIO), University of Florence, 50121 Florence, Italy; marco.zaccaroni@unifi.it; 4 Parco Nazionale Arcipelago Toscano, Loc. Enfola, 57037 Portoferraio, Italy; giannini@islepark.it

**Keywords:** southern European brown hare, diet, micro-histological analysis, feeding preferences, dietary plasticity

## Abstract

This study investigates the feeding behavior of the European brown hare on Pianosa Island, Italy, focusing on seasonal dietary preferences in coastal and inland areas. We quantified plant species frequencies in vegetation and fecal pellets, assessed α and β diversity, compared diets via Bray–Curtis matrices, and evaluated selection preferences using the Resource Selection Ratio. During spring, the coastal diet was predominantly composed of grasses, particularly *Dactylis glomerata*, while non-leguminous forbs like *Hedypnois rhagadioloides* were prominent inland. Fabaceae, including *Lotus ornithopodioides*, were highly preferred. In autumn, non-leguminous forbs, such as *Crepis foetida*, were favored on the coast, while grasses like *Bromus hordeaceus* dominated inland. Fabaceae, particularly *Trifolium* species, remained a key component of the diet. Plant diversity peaked in spring and decreased markedly in autumn. Dietary overlap between seasons was moderate, with higher similarity observed in the coastal area. The study underscores the importance of Fabaceae and seasonal vegetation changes in shaping the hare’s diet. These findings provide valuable insights for conservation, emphasizing the need to preserve key plant species and habitats to support isolated hare populations in Mediterranean ecosystems.

## Simple Summary

This study examines the feeding behavior of the European brown hare on Pianosa Island, Italy, across seasons and habitats. Coastal areas had fewer plant species than inland areas, with grasses dominating the diet in spring and herbaceous plants with large leaves in autumn. Legumes were highly preferred year-round. Plant species diversity was higher in spring but declined significantly in autumn. The hare exhibited dietary flexibility, relying entirely on herbaceous plants and avoiding shrubs, likely due to their scarcity and the absence of feeding competitors. These findings highlight the hare’s adaptability to seasonal changes and emphasize the importance of preserving key plant species for its survival in Mediterranean ecosystems.

## 1. Introduction

The European brown hare *Lepus europaeus*, widely distributed across Europe, has experienced extensive translocations and hybridization, often compromising the genetic integrity of native populations [1,2,3]. Among these, the taxonomic classification of subspecies remains contentious. For instance, *L. europaeus meridiei*, described by Hilzheimer [4] based on limited morphological data and a small number of specimens [5], is not recognized by Smith et al. [1]. In contrast, Mengoni et al. [6] identified the hare population on Pianosa Island as belonging to the subspecies *L. e. meridiei*, supported by genetic evidence showing a unique ancestral mitochondrial haplotype (Leu2) and distinct skull morphology [7]. These findings align with earlier studies documenting the prevalence of ancestral haplotypes in isolated Italian populations, likely preserved since the last glaciation [8,9,10]. However, the absence of genetic comparisons with historical specimens of *L. e. meridiei* prevents definitive conclusions about the validity of this subspecies. While this debate [6,7] highlights the complexity of subspecies classification within the genus *Lepus*, it is not the primary focus of this study. Instead, our research emphasizes the ecological significance of the Pianosa hare population (*L. europaeus*, Leu2 Haplotype), which remains unaffected by recent translocations and is vulnerable to hybridization and habitat changes, with a particular focus on its feeding behavior.

With the vegetation on many Mediterranean areas gradually progressing toward a climax state typical of Mediterranean scrubland [11], and in the absence of management interventions, the habitat’s suitability for hares is expected to decline further over the coming decades. The most recent comprehensive census, conducted under a LIFE Project [12], indicates a population density of around 10 hares/km^2^. In light of these habitat variations, the study of feeding behavior plays a critical role in understanding and conserving isolated populations. Feeding patterns not only reveal dietary adaptations to specific ecosystems but also provide insights into habitat preferences and resource dependencies; this is observed across different species in various habitats [13]. Numerous studies on the E. brown hare [14,15,16,17,18] have demonstrated a strong correlation between feeding behavior and seasonal habitat use, highlighting the need for habitat-specific management strategies [19]. Similar findings have emerged from Mediterranean environments, particularly regarding the Italian hare *L. corsicanus* [20,21,22,23], where studies also highlight the relationship between dietary plasticity and ecological resilience [24]. Comparable patterns have been observed worldwide for other hare species such as *L. granatensis* [25], *L. timidus hibernicus* [26], *L. arcticus* [27], *L. californicus* [28,29], *L. flavigularis* [30], *L. americanus* [31] and *L. starki* [32]. These findings emphasize the importance of feeding behavior as a key factor in designing effective conservation interventions, highlighting that an understanding of the nutritional ecology of herbivores is essential for wildlife management, conservation, and maintaining functional ecosystems [33]. The confined population of hares on Pianosa Island provides a unique opportunity to study feeding ecology in a typical Mediterranean environment. Unlike mainland populations, the hares on Pianosa face no competition from other herbivores, allowing for a direct assessment of their feeding preferences. The absence of natural predators creates a controlled environment that facilitates the observation of undisturbed behavioral patterns. In our study system, feral cats represented the sole source of predation risk for hares prior to their 2018 eradication (see Section 4.3 for detailed analysis). In contrast, interspecific interactions and niche partitioning typically shape animal communities, with predators optimizing hunting strategies, prey adopting antipredator tactics, and competitors displaying behavioral plasticity to minimize conflicts and meet life-history needs [34,35].

In the present study, the first conducted on the feeding preferences of this hare population, we evaluated the effect of season (Spring, Autumn) separately in two areas (internal and coastal) on diet composition and feeding selection. Specifically, we aimed to explore its dietary ecology by: (1) analyzing diet composition; (2) describing the use and seasonal selection of plant resources; (3) identifying key dietary plant species; and (4) evaluating dietary differences between periods in internal and coastal environments.

## 2. Materials and Methods

### 2.1. Study Area

**Figure 1:**
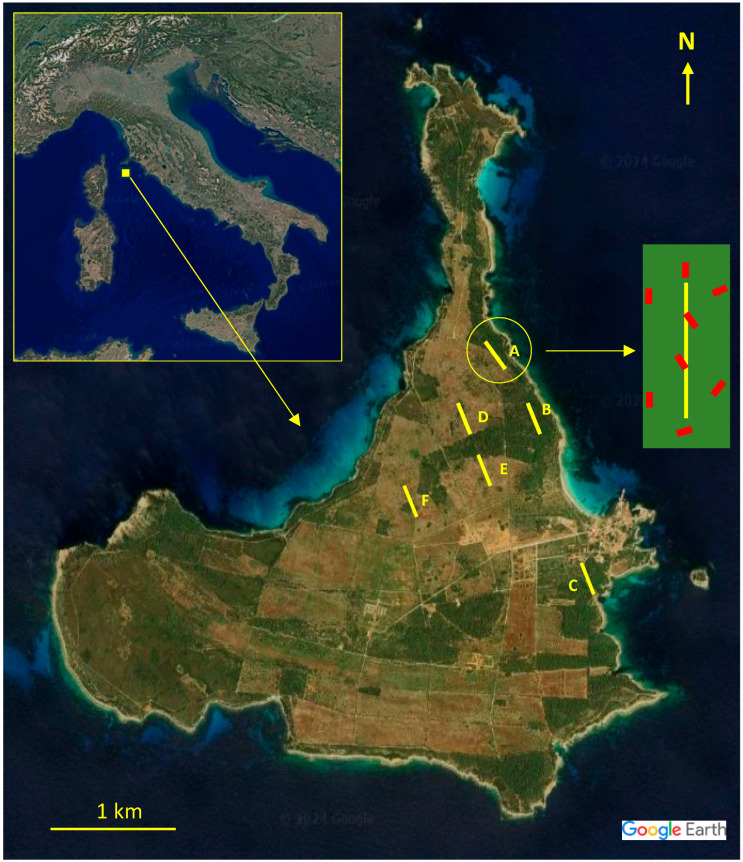
Satellite images of Italy and Pianosa island in detail [37]: (A, B, C) vegetation coastal transects; and (D, E, F) vegetation internal transects. Red stripes depict an example of the positioning of the eight pellet transects (2 m × 30 m) in a vegetation transect (1 × 300 m).

Pianosa Island, Tuscany, Italy (42°34′12″ N, 10°05′32″ E) is a protected area that covers about 10.3 km^2^ (Figure 1). Annual temperature and precipitation means in 2022, the year of the reliefs, were +15.8 °C and 497 mm, respectively. The macro-bioclimate is Mediterranean, mostly meso-Mediterranean bioclimatic belt [36]. A maximum-security prison was located on the island until 1997; it closed permanently after the last inmate was transferred and almost all staff and their families left the island. Currently, the island has around fifteen residents (all parolees) and 4–5 guards (Carabinieri and Prison officers).

The natural vegetation of Pianosa consists primarily of evergreen sclerophyll shrublands and woodlands. Historically confined to a narrow coastal strip around cultivated and grazed central areas, these regions are now undergoing various stages of shrub and tree recolonization. Inland forests are mostly pine plantations, dominated by *Pinus halepensis*, along with *Pinus pinea* and *Pinus pinaster*, often with a significant shrub layer. *Quercus ilex*, once limited to small clusters, has recently spread rapidly across abandoned fields and pine forests, thriving in sheltered areas that are less exposed to wind. Coastal shrublands include *Juniperus turbinata* and *Pistacia lentiscus*, with other shrubs like *Rhamnus alaternus*, and halophytes such as *Cakile maritima*, *Crithmum maritimum*, and *Aster tripolium*. Sandy soils support vegetation adapted to saline, windy conditions like *Eryngium* spp. and *Limonium* spp. Low shrub formations, dominated by *Rosmarinus officinalis*, *Pistacia lentiscus*, and *Cistus monspeliensis*, are widespread, especially in the southwestern part. Rocky coastal areas feature garrigues dominated by *Helichrysum litoreum*. While agricultural activity has diminished due to protection, remnants of olive groves and vineyards persist. Old olive trees and wild olives (*Olea europaea* var. *sylvestris*) thrive in abandoned areas. Previously cultivated fields were overtaken by perennial grasslands and are now progressively colonized by shrubs and trees. The total land area in Pianosa that is categorized as suitable for hares, based on data from the recent LIFE Project [12], is 930.05 ha. This is subdivided into the following land cover categories: fine forest/scrubland (261.18 ha); former cultivated land with scrub and shrubland (512.71 ha); former cultivated land/grassland (145.66 ha); and mowed olive grove (10.50 ha). Pianosa island is entirely included within the Special Protection Area (SPA) IT5160016 and the Site of Community Importance (SCI) IT5160013. It is of significant ornithological value, as it is not only an important stopover site for migratory birds (e.g., *Streptopelia turtur*, *Ciconia ciconia*, *Apus pallidus*, *Phoenicurus phoenicurus*) but also hosts breeding populations of various bird species of community interest, such as *Lanius minor*, *Caprimulgus europaeus*, and *Calandrella brachydactyla*, as well as numerous pairs of *Calonectris diomedea* and *Ichthyaetus audouinii*. Apart from hares, three small mammals populate this island: *Suncus etruscus, Mus musculus*, and *Apodemus sylvaticus*. In 2018, as part of the aforementioned LIFE Project, a population of 53 free-ranging cats was eradicated through repeated capture sessions, as they posed a serious threat to the survival of many native species. Indeed, island fauna—particularly bird species that nest on islands or use them as stopover sites during migration—are especially vulnerable to predation by feral cats [38,39,40,41]. The feral cat colony originated from cats abandoned by personnel and their families when they left Pianosa. Similarly, the Black rat *Rattus rattus*, by far the most widespread species of its genus on Mediterranean islands, has been targeted for eradication, as well as on the islands of Giannutri and Montecristo in the same archipelago [42,43]. In addition, more than 3500 pheasants (*Phasianus colchicus*) and 300 hybrid partridges (*Alectoris* spp.)—game species originally introduced to create a hunting reserve during the island’s time as a maximum-security prison—were removed from Pianosa, along with 150 European hedgehogs (*Erinaceus europaeus*). The Tuscan Archipelago National Park authorities eliminated these non-native species as part of their conservation management program.

### 2.2. Plant Sampling

To evaluate the relative frequencies of plant species, 6 permanent transects (A, B, C coastal and D, E, F internal) were established (Figure 1). Sampling occurred during in spring (April) and autumn (October). Each transect was 300 m long and placed to encompass all vegetation types present in the study areas, with a minimum spacing of 200 m between transects. The quadrat method [23] was employed for plant frequency assessments: 150 quadrats of 1 m^2^ were sampled per transect, with systematic skipping of the subsequent quadrats. Plant species were classified into four vegetation forms: grasses (G) including graminoids, leguminous forbs (LF), non-leguminous forbs (NLF), and shrubs (S). The taxonomic nomenclature followed Bartolucci et al. [44]. A specimen of each observed species was collected from the transects and processed according to Maia et al. [45]. To create a reference collection for each species, histological fragments of each anatomical part were photographed under light microscopy and catalogued using the Leica Q500IW image analyzer (Leica Imaging System Ltd., Cambridge, UK). Plant species, families, and vegetation forms were used to calculate their relative frequencies.

### 2.3. Fecal Pellet Sampling and Analysis Procedure

Fecal pellet sampling was conducted seasonally during spring and autumn to align with peak plant phenological stages [23]. At each study site, we systematically established eight parallel transects (2 m width × 30 m length), maintaining a minimum 100 m inter-transect distance to ensure the spatial independence of animal sampling [23,25]. This design minimized the probability of repeated sampling of individual animals while maintaining vegetation sampling representativeness. The positioning of pellet transects, in accordance with these conditions, was adapted to the specific characteristics of each site (Figure 1). Only fresh fecal pellets (bright brown in color) were collected. For each collection, at least six pellets of varying sizes and shapes were combined to create a single composite sample. A total of 12 composites samples were analyzed (2 periods × 6 sites). Pellets were processed following Freschi et al. [24,46]. For each composite sample, 30 microscope slides were prepared and analyzed using the Leica Q500IW image analyzer obtaining 200 readings for each sample, counting non-overlapping plant fragments in systematic transepts across a slide along alternate rows. Plant species identification relied on comparing epidermal cell features and structures (e.g., stomata, trichomes) to the reference collection, which was updated continuously with plant material from the study site. Microphotographs from all *taxa*/structures were made with the same magnification to facilitate a fast comparison between the reference collection and the fecal material [20]. The reference collection is housed at the Laboratory of Environmental and Applied Botany, University of Basilicata. Unidentified fragments (7.3%) were excluded from further analysis.

### 2.4. Statistical Analysis

Plant species, families, and vegetation forms were used to calculate their relative frequencies. For the fecal analysis, the relative frequencies of plant species were determined by dividing the total number of fragments attributed to a specific taxon by the total number of identified fragments for each season, following established protocols [21,23].

To assess the diversity of plant species in the vegetation and diet, the following alpha diversity indices were computed: (a) the Shannon Diversity Index (*H*) measures species diversity, with typical values ranging between 1.5 and 3.5 and rarely exceeding 4 [47]; (b) the Margalef Index (*D*) evaluates species richness, where higher values indicate greater richness [48,49]; and (c) Buzas and Gibson’s Evenness Index (*E*) assesses the evenness of species distribution within the diet [50]. Differences in diversity indices between DS and WS were tested using Student’s t-test. Vegetation similarity between spring and autumn was quantified by the Chao Similarity Index [51] and Sørensen Similarity Index [52], varying between 0 (no similarity) and 1 (complete similarity). Dietary differences were analyzed using Bray–Curtis similarity matrices computed from averaged dietary composition data [53].

Diet selection was assessed for vegetation forms and plant families using the Resource Selection Ratio (*w_i_*):

*w_i_* = *o_i_*/*p_i_*

where *o_i_* is the proportion of a botanical family or life form in the diet and *p_i_* is its available proportion [54]. Based on this index, feeding behavior related to a plant species/family was classified as: *w_i_* > 1.1, Preference (P); 0.9 < *w_i_* < 1.1, Indifference (I); and *w_i_* < 0.9, Avoidance (A). The choice to use a range for indifference instead of a single value (*w_i_* = 1), in our opinion, reflects a more biologically realistic interpretation of the collected data on feeding behavior. Differences in selection ratios were tested using a χ^2^ test [55]. All statistical analyses were performed using R software (version 3.6.1.; R Core Team) [56].

## 3. Results

### 3.1. Vegetation and Dietary Botanical Composition

The number of plant species identified along the transects is markedly lower in the coastal area (68 in spring and 36 in autumn) compared to the internal area (83 in spring and 54 in autumn) (Table 1). This mirrors a wider choice of utilized plants in the diet in the internal area compared to the coast, as well as the greater variety of plant species utilized in spring rather than in autumn, which is a key focus of this study.

**Figure 2:**
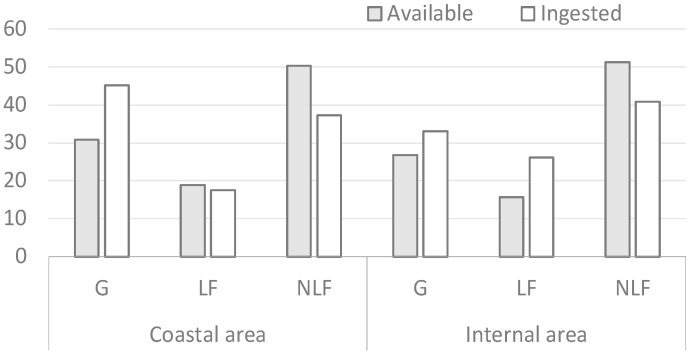
Percentage contribution of the vegetation forms in spring.

The percentage contributions of life forms to both vegetation and diet composition during spring are shown in Figure 2. Grasses (G) are the most important vegetation form in the diet during spring in the coastal area, with a notable preference for this life form, followed by non-leguminous forbs (NLF) and leguminous forbs (LF). In the inland area, NLF were the most ingested, even if not preferred, followed by G and LF, which were the selected life forms.

**Figure 3:**
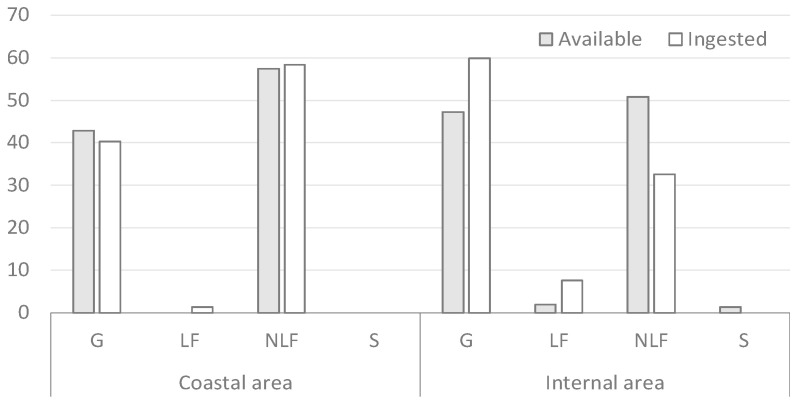
Percentage contribution of the vegetation forms in autumn.

In autumn, NLF were the most selected life form on the coast, followed by G (Figure 3). In the inland area, the most ingested life form was G, with a marked positive selection, followed by NLF and LF. For LF, high selection must be emphasized (Figure 3).

In spring, along the coastal area (Table S1), *Triticum vagans* (7.72%) and *Anisantha madritensis* (7.32%) are the most ingested species, while *Dactylis glomerata glomerata* (3.63%) and *Festuca myuros* (4.26%) are among the most available. In the internal area, *Bromus hordeaceus* (5.22%) and *Medicago truncatula* (3.81%) are highly ingested, with *F. myuros* ssp. *myuros* (3.62%) and *Lathyrus sativus* (2.2%) being the most available. Grasses, like *D. glomerata* and *F. myuros*, dominate in both areas, while LF such as *M. truncatula* and *L. sativus* play a significant role in the internal zone. During autumn (Table S2), in the coastal area, *D. glomerata* ssp. *glomerata* is the most abundant species (11.67%) and also the most often ingested (14.8%), followed by *Allium commutatum* (10.93%) and *Lagurus ovatus* (8.58%). In the internal area, *F. myuros* ssp. *myuros* dominates in availability (8.85%), while *B. hordeaceus* is the most ingested species (12.76%). These results highlight the importance of grasses, like *D. glomerata* and *F. myuros* ssp. *Myuros*, in both areas, with *B. hordeaceus* being particularly favored in the internal zone.

### 3.2. Botanical Diversity and Similarity in Vegetation and Diet

The Shannon and Margalef diversity indices are significantly higher in spring than in autumn at both the internal and coastal sites (Table 2). The internal site shows greater vegetation similarity between seasons compared to the coastal site, with higher values for both the Chao Similarity Index (0.712 vs. 0.675) and the Sorensen Index (0.724 vs. 0.683).

A marked and significant decrease is observed in α-diversity across all indices from spring to autumn in both sites (Table 3). Specifically, the Shannon Index declines at the internal site (from 2.900 ± 0.050 to 2.408 ± 0.074, *p* = 0.011,) and the coastal site (from 2.750 ± 0.087 to 2.300 ± 0.087, *p* = 0.021,). Similarly, the Margalef Index shows a reduction at the internal (from 3.825 ± 0.075 to 3.208 ± 0.008, *p* = 0.001) and coastal sites (from 3.700 ± 0.110 to 3.083 ± 0.101, *p* = 0.016,). The Buzas and Gibson Index also decreases at both the internal (0.865 ± 0.005 to 0.795 ± 0.009, *p* = 0.021) and coastal sites (0.840 ± 0.024 to 0.780 ± 0.012, *p* = 0.021). Chao Index values were 0.712 for the internal site and 0.675 for the coastal site, while Sorensen Index values were 0.724 for the internal site and 0.684 for the coastal site.

### 3.3. Dietary Selectivity

In the coastal area during spring (Table 4), several plant families are significantly preferred in the diet (*p* < 0.05). Among these, Fabaceae stand out as highly preferred (*w_i_* = 1.265), alongside Apiaceae (*w_i_* = 7.731) and Lamiaceae (*w_i_* = 3.480). Brassicaceae (*w_i_* = 2.441 and Poaceae (Wi = 1.468) are also favored, although less prominently. In contrast, families like Convolvulaceae (*w_i_* = 0.015) and Euphorbiaceae (*w_i_* = 0.088) are avoided. In autumn, the preference for Fabaceae becomes even more pronounced, making it the most favored family, albeit with low statistical significance (*w_i_* = 13.800, *p* = 0.148). Other significantly preferred families (*p* < 0.05) include Amaryllidaceae (*w_i_* = 2.810), Apiaceae (*w_i_* = 5.579), and Rosaceae (*w_i_* = 6.575). Conversely, Poaceae (*w_i_* = 0.832) are slightly less preferred.

Distinct patterns were observed between spring and autumn in the internal area (Table 5). In spring, the most preferred families (*p* < 0.05) include Malvaceae (*w_i_* = 2.385), Asteraceae (*w_i_* = 1.446), and Fabaceae (*w_i_* = 1.37), indicating their importance in the diet during this season. Families, like Poaceae (*w_i_* = 1.205), are also preferred, though to a lesser extent. In contrast, several families, such as Euphorbiaceae (*w_i_* = 0.048), Asphodelaceae (*w_i_* = 0.826), and Brassicaceae (*w_i_* = 0.843), are avoided, reflecting their lower palatability or availability. In autumn, the preference for Amaryllidaceae (*w_i_* = 8.167) becomes highly pronounced, making it the most favored family. Other significantly preferred families (*p* < 0.05) include Fabaceae (*w_i_* = 3.842), Asparagaceae (*w_i_* = 3.6), and Caryophyllaceae (*w_i_* = 2.452). On the other hand, families, like Asphodelaceae (*w_i_* = 0.021), and Boraginaceae (*w_i_* = 0.648), are consistently avoided, indicating that they are less desirable or accessible during this season.

**Figure 4:**
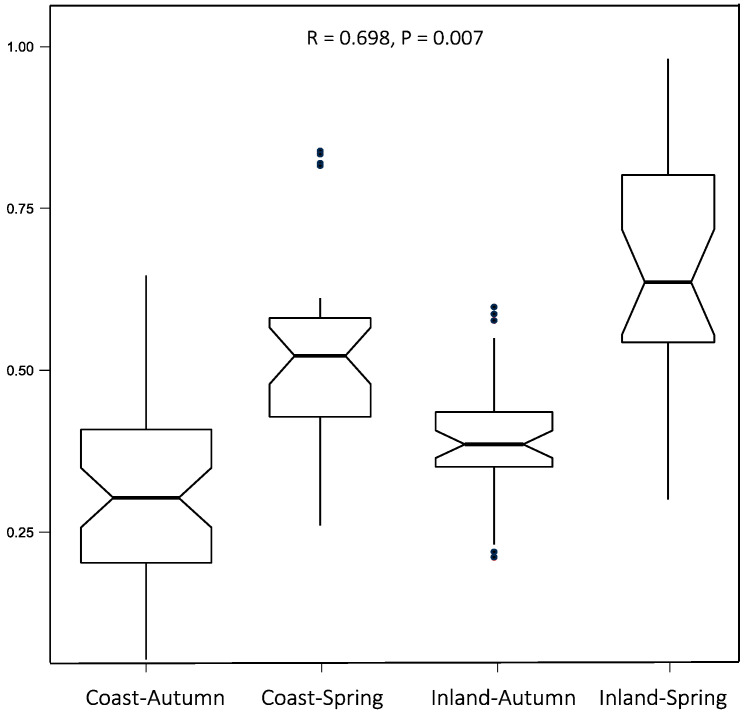
Analysis of similarities of diet between the coastal and internal environments during spring and autumn.

Similar diets occurred in both environments (Figure 4). The correlation coefficient (R = 0.698) indicates a moderately strong positive relationship that is statistically significant (P = 0.007). However, the analysis also reveals distinct differences that appear to be strongly influenced by seasonal factors.

## 4. Discussion

This study revealed several important findings about the Pianosa hare’s feeding ecology. Even within this limited study area, we observed distinct differences in vegetation form consumption between the coastal and internal sites. Seasonal dietary shifts were particularly notable. In spring, grasses (G) dominated the coastal diet while non-leguminous forbs (NLF) prevailed inland; however, this pattern was reversed in autumn, when grasses became predominant at the internal sites. The hares demonstrated flexible foraging behavior, adjusting their diet composition according to food availability. Their diet consisted exclusively of herbaceous plants, with no evidence of shrub consumption. Notably, the internal site maintained slightly higher plant diversity and richness across both seasons, likely due to the more stable environmental conditions that appear to be better-suited to supporting the hares’ nutritional and habitat requirements.

### 4.1. Biodiversity and Seasonal Impact

This study area exhibited a markedly lower diversity of botanical species compared to other Mediterranean environments previously investigated for the feeding behavior of *L. corsicanus*, such as coastal sites in Latium and Corsica [23,24], as well as for *Capreolus capreolus italicus* [57]. Specifically, 89 species were recorded in Pianosa (including both species unique to the area and those shared between the two environments), compared to over 130 species in Corsica and about 230 along the Latium coast. Despite the low variety of species, the results reveal a consistent pattern of higher biodiversity in spring compared to autumn for both internal and coastal sites, as measured by α-diversity indices. The higher diversity and richness observed at the internal sites across both seasons align with findings from Rizzardini et al. [23] conducted at Corsican coastal sites at a comparable latitude to Pianosa Island. These authors [20] showed that the highest species richness was also found in areas less influenced by dynamic factors such as salinity fluctuations, wind exposure, and human activity. These factors create a more challenging environment for species persistence, further highlighting the importance of stable habitats for biodiversity. To address potential incomplete sampling, we employed the Chao Index to estimate total species richness, alongside the Sørensen Index. The Chao Index better accounts for rare species and provides a more accurate estimate [51], which is especially relevant for small areas like Pianosa Island. Nevertheless, both indices have indicated slightly higher diversity and stability at the internal site, although the small differences suggest significant species overlap between sites. With regard to *α* diversity, while species richness and diversity decline in autumn, stable evenness indicates a consistent distribution of individuals among available plant species. This condition facilitates a more stable diet composition, supported by the hare’s ability to adapt to seasonal changes through the balanced use of remaining food resources [25,26,58,59]. Such flexibility likely enhances the hare’s resilience in an environment characterized by limited and variable food availability. However, the coastal site shows more pronounced seasonal changes in evenness, reflecting greater variability in species distribution and abundance compared to the internal site. Similarity indices reveal moderate similarity in species composition between spring and autumn for both sites. The internal site shows slightly higher similarity compared to the coastal site, indicating greater species turnover in the coastal area due to its exposure to more variable environmental conditions. In contrast, the internal site, being more sheltered, maintains a more stable species composition over time, making it the most suitable habitat for the hare.

### 4.2. Feeding Selection

Across both spring and autumn seasons, Poaceae dominates vegetation availability, with species like *F. myuros*, *D. glomerata*, and *Cynosurus echinatus* being particularly abundant. In the diet, grasses, such as *T. vagans*, *Avena barbata*, and *B. hordeaceus*, are heavily ingested, highlighting their importance as a food source. Plants from this family constitute a significant portion of the diet in several *Lepus* species, including *L. europaeus* [14,17,60,61,62,63], *L. timidus hibernicus* [26,64,65], *L. arcticus* [27], *L. californicus* [28,29,66,67], *L. flavigularis* [30], *L. granatensis* [25], and *L. starcki* [32]. According to Castellaro et al. [68], the high contribution of Poaceae in the diet is due to their wide distribution throughout the year, good palatability, and high cellulose content, which provides valuable energy reserves, especially during colder periods. This pattern underscores the hare’s preference for grasses and its ability to adapt foraging behavior to seasonal changes in resource availability. Non-leguminous forbs, including *Raphanus raphanistrum*, *Crepis foetida*, and *Plantago lanceolata*, are also selectively consumed despite their lower availability. In autumn, the preference for Fabaceae increased notably in both sampling areas, particularly on the coast, where its selection exceeded 13 times its availability in the vegetation. This strong preference for Fabaceae during this season can be attributed to its higher protein content and lower fiber content compared to grasses, which experience an increase in fiber content during autumn [25,69]. Notably, the high selection rate of *A. commutatum*, particularly observed in autumn, where its contribution to the diet was over three times its presence in vegetation, could be attributed to its succulence and nutritional value. This feeding behavior has also been observed in the Italian hare [19] during the same period, likely due to the high moisture content of its leaves and bulbs, which makes the species particularly preferred in dry periods when water availability is limited. Such selective foraging highlights the hare’s ability to optimize resource utilization in response to environmental conditions. Among the morphological and chemical factors that positively influence plant palatability, Vallentine [70] highlights several key traits: the presence of succulent leaves rather than poor flowering; the absence of thorns; easy accessibility to edible parts; low levels of tannins (which impart a bitter taste); and the absence of toxic alkaloids and glucosides.

### 4.3. Feeding Strategy in Absence of Predation and Competition

Classical foraging theory indicates that organisms optimize their feeding strategies to balance nutritional intake against predation risk. Spatial selection in lagomorphs manifests through the avoidance of high-risk microhabitats, particularly in areas with either excessive exposure (minimal vegetative cover) or excessive predator concealment opportunities (dense thickets) [71]. These behaviors yield constrained dietary breadth and potentially suboptimal nutrient intake due to restricted foraging areas. Given the hares’ rapid generational turnover (7–9 months) and limited lifespan (up to 3 years), we excluded any residual influence of cat predation on their feeding behavior during our 2022 study. We hypothesized negligible behavioral impacts from historical predation pressure. This expectation aligns with two key frameworks: (a) temporal decay of fear effects [72]; and (b) neurobiological reset mechanisms, through which prey species rapidly revert to optimal foraging behaviors post threat [73].

According to some authors [74,75,76], hares benefit from the short vegetation generated by grazing. However, Bakaloudis [77], focusing on *L. europaeus*, and Lankist and Maher [78], focusing on *L. americanus*, argue that this claim is not substantiated, reporting instead that the alteration of vegetation caused by grazing is likely responsible for the low habitat quality, which could result in a reduction in hare populations. In addition, selection may also be influenced by forage biomass or by plant responses to browsing across vegetative phases (e.g., defensive activity of tannins against herbivores) [79,80]. In our case, it is notable that all fragments observed in the Pianosa hare’s diet belong exclusively to herbaceous *taxa*. Numerous studies on the feeding behavior of various *Lepus* species highlight their strong preference for herbaceous plants, which is well-documented in the literature, although they do not exclude shrub and tree species [12,13,22,23,24,25,27,29]. On Pianosa island, shrub species were entirely absent from the hare’s diet. While this could be partially attributed to the limited availability of shrubs in the study area—only three species were present along the transects—it is unlikely to be the sole explanation. In contrast, our previous studies on the Italian hare in Mediterranean environments documented the inclusion of shrubs in their diet, ranging from 9 to 12% along the Latium coast [21] to 1% at Tallone on the Corsican coast [20]. These differences suggest that the presence of competitors and predators in these environments may drive dietary diversification. In our case, the absence of predators—thanks to the above-mentioned recent eradication efforts—and the lack of competitors create favorable conditions for the survival and adaptation of the hare. This is evidenced by its mean population density of 10 hares/km^2^—a satisfying value—despite the limited availability and variety of food species considered. Furthermore, the absence of such pressures likely enables the hare to rely exclusively on herbaceous plants, optimizing its foraging efficiency without the need to exploit less accessible or less preferred resources such as shrubs or trees. Comparative studies highlight that our findings on Pianosa hares represent a unique intermediate scenario. While continental European hares maintain broader dietary plasticity to exploit seasonally variable resources [74], our results demonstrate how insular conditions shape dietary ecology, providing insights for conservation strategies for isolated hare populations, though local vegetation and climate remain primary factors. The unique combination of insular dietary specialization with persistent behavioral plasticity suggests that Pianosa hares have developed an optimal foraging strategy tailored to their intermediate ecological context; it is an island sufficiently large to sustain stable populations but small enough to generate strong selective pressures. This pattern reflects an evolutionary trade-off between the energetic efficiency of dietary specialization and the risk-mitigation advantages of trophic plasticity—a balance that may become increasingly precarious under climate change, as shifting plant phenology threatens to disrupt these finely tuned adaptations.

## 5. Conclusions

This study demonstrates that the European brown hare on this island exhibits significant dietary plasticity, adapting its feeding behavior to seasonal changes in plant availability. Coastal and internal areas differ in plant diversity, with the internal area providing a more stable and diverse food supply over time. The observed dietary specialization underscores the critical role of ecological context in shaping foraging behavior, particularly in isolated environments with unique resource availability and competition dynamics. The Pianosa hare’s reliance on herbaceous plants highlights how the absence of interspecific competition can lead to niche simplification, enabling efficient exploitation of the most abundant resources. These findings contribute to a broader understanding of resource partitioning and dietary adaptation in low-competition environments. Future research should explore the long-term impacts of habitat variations on the dietary habits and survival of this isolated hare population. Investigating the adaptations linked to dietary plasticity could provide deeper insights into responses to environmental constraints. Additionally, comparative studies with other island populations or mainland hares would help to clarify the role of isolation in shaping foraging strategies. Monitoring population dynamics alongside food resource fluctuations could further elucidate the resilience of this species in the face of ecological shifts.

## Figures and Tables

**Table 1:** Plant species in vegetation and diet.

Total	Spring		Autumn	
	Coast	Internal	Coast	Internal
Vegetation	68	83	36	54
Diet (a + b)	39	56	29	36
a, selected	29	33	19	23
b, not selected	10	23	10	22

**Table 2:** Vegetation biodiversity indices (mean ± SE) in spring and in autumn at the internal and external sites.

Index	Internal Site			Coastal Site		
	Spring	Autumn	*p*	Spring	Autumn	*p*
	Diversity (α diversity)					
Shannon, H	3.494 ± 0.053	2.758 ± 0.099	0.013	3.434 ± 0.006	2.585 ± 0.166	0.002
Margalef	7.418 ± 0.703	4.026 ± 0.152	0.001	6.327 ± 0.628	4.010 ± 0.387	0.009
Buzas and Gibson, E	0.832 ± 0.08	0.797 ± 0.009	0.931	0.852 ± 0.084	0.789 ± 0.009	0.789
	Similarity (β diversity)					
Chao	0.712			0.675		
Sørensen	0.724			0.683		

**Table 3:** Diet biodiversity indices (mean ± SE) in spring and autumn at the internal and external sites.

Index	Internal Site			Coastal Site		
	Spring	Autumn	*p*	Spring	Autumn	*p*
	Diversity (α diversity)					
Shannon, H	2.900 ± 0.050	2.408 ± 0.074	0.011	2.750 ± 0.087	2.300 ± 0.087	0.021
Margalef	3.825 ± 0.075	3.208 ± 0.008	0.001	3.700 ± 0.110	3.083 ± 0.101	0.016
Buzas and Gibson, E	0.865 ± 0.005	0.795 ± 0.009	0.021	0.840 ± 0.024	0.780 ± 0.012	0.021
	Similarity (β diversity)					
Sørensen	0.56			0.62		

**Table 4:** Selection ratio (*w_i_*) for botanical families in the coastal area.

Family	Spring			Autumn		
	*w_i_*	Feeding Behavior	*p*-Value	*w_i_*	Feeding Behavior	*p*-Value
Amaryllidaceae	4.460	P	0.159	2.810	P	0.034
Anacardiaceae	-	-	-	2.000	P	0.371
Apiaceae	7.731	P	0.045	5.579	P	0.000
Asparagaceae	0.159	A	0.124	0.128	A	0.261
Asphodelaceae	-	-	-	0.043	A	0.136
Asteraceae	0.914	I	0.031	1.936	P	0.037
Boraginaceae	0.400	A	0.227	0.385	A	0.529
Brassicaceae	2.441	P	0.006	0.256	A	0.317
Caryophyllaceae	2.248	P	0.085	0.612	A	0.064
Convolvulaceae	0.015	A	0.048	0.057	A	0.051
Dipsacaceae	1.139	P	0.038	2.128	P	0.177
Euphorbiaceae	0.088	A	0.009	0.163	A	0.042
Fabaceae	1.265	P	0.000	13.80	P	0.148
Gentianaceae	0.080	A	0.446	-	-	-
Geraniaceae	1.000	I	0.066	0.163	A	0.296
Lamiaceae	3.480	P	0.036	0.175	A	0.045
Linaceae	0.036	A	0.519	-	-	-
Malvaceae	0.067	A	0.221	-	-	-
Orobanchaceae	0.033	A	0.077	-	-	-
Plantaginaceae	2.007	P	0.004	2.164	P	0.688
Poaceae	1.468	P	0.005	0.832	A	0.006
Primulaceae	0.067	A	0.049	-	-	-
Resedaceae	0.200	A	0.078	-	-	-
Rosaceae	-	-	-	6.575	P	0.038
Rubiaceae	0.057	A	0.050	-	-	-

Feeding behavior: (P) preference, (I) indifference, and (A) avoidance.

**Table 5:** Selection ratio (*w_i_*) on botanical families in the internal area.

Family	Spring			Autumn		
	*w_i_*	Feeding Behavior	*p*-Value	*w_i_*	Feeding Behavior	*p*-Value
Amaryllidaceae	-	-	-	8.167	P	0.015
Anacardiaceae	-	-	-	0.179	A	0.428
Apiaceae	0.371	P	0.077	0.673	A	0.023
Asparagaceae	-	-	-	3.6	P	0.042
Asphodelaceae	0.826	A	0.063	0.021	A	0.033
Asteraceae	1.446	P	0.002	1.343	P	0.002
Boraginaceae	1.359	P	0.048	0.648	A	0.442
Brassicaceae	0.843	A	0.070	0.087	A	0.471
Caprifoliaceae	0.2	A	0.352	-	-	-
Caryophyllaceae	0.59	A	0.003	2.452	P	0.055
Convolvulaceae	0.024	A	0.056	0.192	A	0.066
Dipsacaceae	-	-	-	1.197	P	0.336
Euphorbiaceae	0.048	A	0.053	1.091	P	0.098
Fabaceae	1.37	P	0.000	3.842	P	0.072
Geraniaceae	0.266	A	0.083	-	-	-
Lamiaceae	0.696	A		0.06	A	0.662
Linaceae	0.086	A	0.228	-	-	-
Malvaceae	2.385	P	0.154	-	-	-
Oleaceae	-	-	-	0.179	A	0.044
Orobanchaceae	0.24	A	0.062	-	-	-
Papaveraceae	0.769	A	0.088	-	-	-
Plantaginaceae	0.723	A	0.035	1.244	P	0.572
Poaceae	1.205	P	0.017	1.342	P	0
Primulaceae	0.045	A	0.003	-	-	-
Resedaceae	0.154	A	0.255	-	-	-
Rosaceae	0.035	A	0.077	0.161	A	0.05
Rubiaceae	0.043	A	0.026	-	-	-
Scrophulariaceae	0.769	A	0.058	-	-	-
Thymelaeaceae	0.385	A	0.216	0.357	A	0.077

Feeding behavior: (P) preference, (A) avoidance.

## Data Availability

The plant reference collection is available at the Laboratory of Environmental and Applied Botany, University of Basilicata.

## Supplementary Materials

The following supporting information can be downloaded at: https://www.mdpi.com/article/10.3390/vetsci12060546/s1, Table S1: spring: frequencies of plant species, families and life forms in the vegetation and diet (* = plants with evident hare’s bit). Table S2: autumn: frequencies of plant species, families and life forms in the vegetation and diet (* = plants with evident hare’s bit).
