# Supplementary material for: Feeding Behavior of the European Brown Hare (Lepus europaeus, Leu2 Haplotype) on Pianosa Island: Insights into the Absence of Trophic Competition
Source: Vet Sci. 2025 Jun 3;12(6):546. doi: 10.3390/vetsci12060546 (PMC12197477; doi:10.3390/vetsci12060546)
Supplement: Supplementary file 1 [file vetsci-12-00546-s001.zip › vetsci-3557301-supplementary.pdf]

Table S1. Spring: Frequencies of Plant species, Families and Life forms in the vegetation and diet (* = plants with evident hare’s bit).

Life form	Family	Plant species	Coastal area		Internal area	
			Available	Ingested	Available	Ingested
Grasses	Amaryllidaceae	*Allium commutatum* *	0.63	2.81	0	0
	Asparagaceae	*Muscari comosum*	0.63	0	0	0
	Asphodelaceae	*Asphodelus fistulosus* *	0	0	0.52	1.4
		Asphodelus ramosus ssp. ramosus	0	0	1.55	0.31
	Poaceae	*Anisantha madritensis**	3.76	7.32	4.26	2.1
		*Avena barbata**	3.13	6.12	2.33	6.46
		Briza minor	0.63	3.01	0.13	4.98
		Bromus hordeaceus *	1.75	3.11	3.23	5.22
		Catapodium rigidum	0.38	0	0	0
		*Dactylis glomerata ssp. glomerata**	3.63	3.11	0.39	6.15
		*Festuca myuros ssp. myuros**	4.26	5.02	3.62	2.49
		*Holcus lanatus ssp. lanatus**	3.63	0	0.52	0.55
		Hordeum bulbosum	0	0	0.78	0
		Lagurus ovatus ssp. ovatus	0	0	2.58	0
		Hordeum bulbosum	0.63	0.7	0.1	0.47
		*Lagurus ovatus**	0.1	1.91	2.58	0.86
		Lolium multiflorum	3.63	3.01	0.13	1.56
		Setaria italica ssp. viridis	0	0	1.68	0.47
		Trachynia distachya	2.88	1.4	2.45	0
		Triticum vagans	1.25	7.72	0	0
Leguminous forbs	Fabaceae	*Astragalus hamosus**	0	1.3	1.16	1.4
		Astragalus pelecinus ssp. pelecinus	0	0	0.65	1.01
		Coronilla scorpioides	1.13	0.4	0	0
		Lathyrus sativus *	0.25	1.3	2.2	2.1
		Lotus edulis	1.38	0	0.1	0.93
		Lotus ornithopodioides *	2.88	6.02	0.1	2.65
		*Medicago praecox**	3.13	0.4	2.45	2.1
		Medicago sativa	0	0	1.16	4.13
		*Medicago truncatula**	3.26	3.81	0.1	1.79
		Ononis ornithopodioides	0	0	0.78	0.47
		Trifolium angustifolium	0.25	0	0.65	0.08
		Trifolium campestre	0	0	0	0.23
		Trifolium echinatum	0	0	0.13	0
		Trifolium fragiferum	0.38	0	0.26	0
		Trifolium pratense	0	0	0.1	2.02
		Trifolium repens *	0	0	1.94	1.25
		Trifolium stellatum	5.14	0.7	0	0
		Vicia benghalensis	0	0	2.29	0
		*Vicia hybrida**	0.88	2.21	0.39	1.64
		*Vicia melanops**	0.13	1.4	2.58	3.04
		Trifolium pratense	0	0	0.1	0.39
		Trifolium repens *	0	0	0	0.93
Non leguminous forbs	Apiaceae	*Daucus carota*	0	0	1.16	0
		Foeniculum vulgare ssp. piperitum	0.13	0	0.52	0.7
		Thapsia garganica ssp. garganica	0.13	2.01	0.26	0.93
		Tordilium apulum	0	0	3.45	0
	Asteraceae	*Anthemis arvensis ssp. incrassata*	1.75	0	1.55	0.55
		Calendula arvernsis	3.13	0	1.94	0
		*Carduus pycnocephalus**	0	0.9	0.13	1.56
		Carlina corymbosa	1.38	0	0	0
		*Crepis foetida ssp. foetida**	1.25	4.01	1.42	2.34
		Crepis neglecta	2.88	0.9	0	0
		Glebionis coronaria	0.88	0.7	0.65	0.31
		Hedypnois rhagadioloides *	1.63	1.1	0.1	5.14
		Helichrysum litoreum	0.13	0	0	0
		*Hyoseris radiata**	0	0.7	0.52	4.21
		*Hypochaeris glabra**	2.63	1.4	2.84	4.21
		*Hypochaeris laevigata**	0	1.2	0	0
		*Hypochoeris achyrophorus**	0	0.4	0	0
		Inula odora	1.13	0	0	0
		Onopordum illyricum	1.13	0	0	0
		Pallenis spinosa	0.13	0	0	0
		*Picris hieracioides**	0	0	0.26	1.71
		*Reichardia picroides**	0.38	0	0.78	4.28
		*Sonchus oleraceus**	0	0	0.52	1.56
		Urospermum picroides *	0.13	2.11	1.29	0.78
	Boraginaceae	*Anchusa undulata ssp. hybrida*	0.25	0	0.13	1.17
		*Echium parviflorum**	0	0	0.9	0.23
	Brassicaceae	*Cardamine sp.*	0	0	0.9	0
		Lobularia maritima	0.38	0	2.2	0.08
		Matthiola tricuspidata	0	0	0.26	0
		*Raphanus raphanistrum**	1	6.42	0.39	1.09
		Sinapis arvensis	1.25	0	0.13	2.1
	Caprifoliaceae	*Sixalix atropurpurea*	0	0	1.55	0.31
	Caryophyllaceae	*Cerastium arvense*	0	0	0.39	0.23
		Silene angustifolia	0.1	0.7	0	0
		Silene vulgaris	1.25	2.11	0	0
	Convolvulaceae	*Convolvulus althaeoides*	0.75	0	0.26	0
		Convolvulus arvensis	2.13	0	1.68	0
		Convolvulus cantabrica	3.63	0	2.2	0
	Dipsacaceae	*Sixalix atropurpurea*	2.38	2.71	0	0
	Euphorbiaceae	*Euphorbia elioscopia*	0.25	0	2.35	0
		Euphorbia segetalis	0.88	0	0	0
	Gentianaceae	*Blackstonia perfoliata*	1.25	0	0	0
	Geraniaceae	*Erodium sp.*	0.75	0	1.39	0
		*Geranium molle**	0.1	1.5	0	0
		Geranium rotundifolium	0.75	0	1.94	0.62
	Lamiaceae	*Micromeria graeca ssp. graeca*	0	0	1.03	0
		Salvia verbenaca	0	0	0.65	2.34
		Stachys romana	0.75	2.61	1.68	0
	Linaceae	*Linum strictum*	2.49	0	1.16	0
	Malvaceae	*Malva sylvestris*	1.5	0	0.13	0.31
	Orobanchaceae	*Bellardia viscosa*	3.01	0	1.29	0.31
	Papaveraceae	*Papaver rhoeas*	0	0	0.13	0
	Plantaginaceae	*Plantago afra ssp. afra*	0.21	0	1.81	0
		*Plantago coronopus**	0.63	0	1.55	1.65
		*Plantago lanceolata**	2.01	5.74	1.81	2.1
	Primulaceae	*Lysimachia arvensis*	1.5	0	2.2	0
	Resedaceae	*Reseda alba*	0.5	0	0.65	0
	Rosaceae	*Potentilla reptans*	0	0	2.42	0
		Poterium sanguisorba	0	0	2.42	0
	Rubiaceae	*Sherardia arvensis*	1.75	0	3.33	0
	Scrophulariaceae	*Verbascum sinuatum*	0	0	0.13	0
	Thymelaeaceae	*Thymelaea hirsuta*	0	0	0.26	0

Table S2. Autumn: Frequencies of Plant species, Families and Life forms in the vegetation and diet (* = plants with evident hare's bit).

Life Form	Family	Species	Coastal area		Internal area	
			Available	Ingested	Available	Ingested
Grasses	Amaryllidaceae	*Allium commutatum* *	3.89	10.93	0.01	1.37
		*Narcissus miniatus**	0	0	0.01	3.61
	Asparagaceae	*Asphodelus microcarpus*	0	0	0.01	0.36
		Asparagus acutifolius	0.78	0	0	0
	Asphodelaceae	*Asphodelus fistulosus* *	1.17	0	3.43	0
		Asphodelus ramosus subsp. ramosus *	1.17	0	1.12	0
	Poaceae	*Prospero autumnale**	0	0	0.01	8.3
		*Anisantha madritensis**	0	0	5.83	2.04
		*Avena barbata**	0	0	2.79	2.7
		Briza maxima	0	0	0.56	0
		Bromus hordeaceus *	0.01	1.24	5.49	12.76
		*Cynodon dactylon**	6.61	0	6.05	5.19
		*Cynosurus echinatus**	8.95	0.41	0	0
		*Dactylis glomerata ssp. glomerata**	11.67	14.8	5.31	10.59
		*Festuca myuros ssp. myuros**	5.45	4.29	8.85	8.04
		*Lagurus ovatus**	3.11	8.58	6.7	4.89
		*Triticum vagans**	0	1.38	0.01	7.53
Leguminous forbs	Fabaceae	*Lotus ornithopodioides**	0	0	1.12	0
		Trifolium angustifolium	0	0	0.84	0
		*Medicago praecox**	0	0	0.01	0.46
		*Vicia melanops**	0	0	0.01	0.41
Non leguminous forbs	Apiaceae	*Daucus carota*	1.95	8.02	5.87	2.24
		Foeniculum vulgare ssp. piperitum	0	0	1.4	1.22
		Thapsia garganica ssp. garganica	0.01	3.04	0.01	0.56
	Araceae	*Arisarum vulgare ssp. vulgare*	0.39	0	0.28	0
	Asteraceae	*Anthemis arvensis ssp. incrassata*	1.17	0.69	0.01	0.61
		Calendula arvensis *	0	0	2.51	1.37
		*Carduus pycnocephalus**	0.78	0.83	0.28	0.56
		Carlina corycombosa	0.39	0	0.84	0
		*Cichorium intybus**	0	0	0.01	0.46
		*Crepis foetida ssp. foetida**	4	9.27	0.01	0.66
		*Crepis neglecta**	0	0.41	0	0
		Dittrichia viscosa	0	0	0.01	0.81
		Hedypnois rhagadioloides *	0	0	0.01	0.25
		Helichrysum litoreum	1	0	0	0
		*Hyoseris radiata**	0	1.24	0	0
		*Hypochoeris achyrophorus**	0	0	1	0
		*Hypochaeris laevigata**	0	0.55	0.01	1.02
		*Pallenis spinosa**	0.39	3.32	0.01	0.46
		*Picris hieracioides**	0	2.23	0	0
		*Reichardia picroides**	3.5	0.97	0.56	1.12
		*Scolymus hispanicus**	0.78	5.81	0.28	1.07
		*Tolpis umbellata**	0.78	0	0.56	0
	Boraginaceae	*Echium parviflorum**	4.67	1.8	1.96	1.27
	Brassicaceae	*Lobularia maritima*	1.17	0	6.98	0.61
	Caryophyllaceae	*Petrorhagia saxifraga*	0.39	2.21	0	0
		Silene vulgaris ssp. angustifolia	3.89	0.41	1.68	4.12
	Convolvulaceae	*Convolvulus arvensis**	5.84	0	5.77	2.04
		*Convolvulus cantabrica**	3.89	0.55	4.37	0
	Dipsacaceae	*Sixalix atropurpurea**	1.56	3.32	3.81	4.78
	Euphorbiaceae	*Mercurialis annua*	0	0	0.84	2.75
		Euphorbia elioscopia	0.78	0	0.84	0
		Mercurialis annua	0	0	0.84	0
	Geraniaceae	*Erodium sp.*	0	0.97	0	0
		*Geranium molle**	4.67	0	0	0
		Geranium rotundifolium	4.67	0.55	0	0
	Lamiaceae	*Salvia verbenaca**	6.23	1.11	1.68	0
		Teucrium sp.	0.39	0	0	0
	Plantaginaceae	*Plantago coronopus**	1.95	0	1.96	0
		*Plantago lanceolata**	1.95	8.44	3.81	3.41
	Rosaceae	*Potentilla reptans*	0	2.63	1.4	0.36
		Poterium sanguisorba	0	0	0.84	0
Shrubs	Anacardiaceae	*Piastacia lentiscus*	0	0	0.56	0
	Oleaceae	*Olea europaea*	0	0	0.56	0
	Thymeleaceae	*Thymelea hirsuta*	0	0	0.28	0
